# The Impact of Limited Housing Opportunities on Formerly Incarcerated People in the Context of Addiction Recovery

**Published:** 2017-02-06

**Authors:** Dina Chavira, Leonard Jason

**Affiliations:** Department of Psychology, DePaul University, Chicago

**Keywords:** Housing, Substance abuse, Formerly incarcerated individuals, Community re-entry, Homelessness

## Abstract

**Background:**

Formerly incarcerated individuals with substance use disorders encounter numerous obstacles following incarceration that threaten their sobriety. Obtaining safe and stable housing is a notoriously difficult task resulting in precarious housing that can increase the likelihood of relapse. The current study examined the relationship between substance use and 11 housing settings in a sample of 211 formerly incarcerated individuals with a history of substance abuse to identify the housing characteristics with the highest risk of use.

**Methods:**

Participants retroactively reported their alcohol and illicit drug consumption as well as their dwelling for the past 180 days using the Timeline Follow-back method. Housing settings were collapsed into four conceptually distinct categories: Regulated, Independent, Precarious, and Homeless.

**Findings:**

Results showed differences in alcohol and drug consumption across categories, with Regulated settings having less alcohol and substance use reported. The remaining settings with less oversight had a similar percentage of individuals endorse substance use; however, the Precarious setting was associated with the highest consumption of drug use.

**Conclusion:**

Formerly incarcerated individuals with a history of substance use problems would likely benefit from housing with some degree of oversight and financial obligation. More resources should be funnelled into programs to help formerly incarcerated individuals with substance use disorders find housing that will facilitate abstinence during community re-entry.

## Introduction

The prevalence of substance use disorders among criminal justice-involved individuals is staggering: 65% of incarcerated people meet criteria for a substance use disorder [1], compared to 8.5 per cent of the general population aged 12 and older [2]. The link between substance abuse and initial and recurrent criminal justice involvement is well-established. Approximately a third of state and a quarter of federal prisoners committed their crime while under the influence of drugs, and nearly 20 per cent of state and federal prisoners committed their crime to fund their drug use [3]. Efforts to reduce substance abuse in correctional populations generally focus on in-prison treatment while in custody and mandated treatment in the community, often overseen through community supervision (i.e., probation and parole). Research findings suggest the most effective programs are intensive and longer-lasting, employ cognitive-behavioural methods, and have multiple components including transitional aftercare [4–9]; however, only 11% of inmates surveyed in 2006 received treatment [1]. This substantial treatment gap is likely a primary reason substance-involved offenders are 67% more likely to recidivate than non-substance involved offenders [10,11].

Maintaining abstinence is a crucial component of successful community reintegration for formerly incarcerated individuals with substance use disorders. Even with treatment during incarceration, the neurobiological, behavioural, and psychological effects of chronic substance use make the process of recovery challenging. Long-lasting disruptions in the structure and functioning of the brain resulting from repeated substance abuse, including neuro-adaptation, alteration of gene expression, neurogenesis, and synaptogenesis, put individuals at risk of relapse long after drug use has ceased [12,13]. The resultant alterations in the brain circuits involved in reward, motivation, learning and memory, inhibitory control, and executive functioning are associated with impaired decision-making, increased compulsive drug use and drug-related behaviors, decreased engagement in beneficial behaviors, and decreased avoidance of risky behaviors [12–15]. Stress and negative affective states have also been found to increase the risk of relapse [16].

In addition to maintaining sobriety, formerly incarcerated people also have to contend with the challenges associated with community re-entry. Of the numerous resources needed during the community re-entry process, obtaining and maintaining suitable housing is arguably the most important [17]. Housing instability can severely compromise the ability to find and sustain employment, maintain justice compliance, and access general and mental healthcare treatment [18,19]. Securing independent housing is notoriously difficult for formerly incarcerated people for many reasons including the scarcity of affordable housing, landlord discrimination, and the strict requirements for federally subsidized housing [20].

Economic hardship also presents a significant barrier to safe and stable housing for most justice-involved individuals. A 2002 national survey found nearly 60% of people in jail reported earning less than $12,000 yearly [21], and a 2004 survey of inmates in state and federal prisons revealed that the median annual income of the inmates prior to incarceration was 41% less than non-incarcerated people of similar ages [22]. Indirect indicators of socioeconomic disadvantage in the criminal justice system include poor educational attainment [22–25], the spatial concentration of crime in impoverished neighbourhoods [25,26], and the proportion of crime (40%) attributable to poverty [27]. Additionally, the loss of economic and social capital during incarceration is likely a major contributing factor to increased residential instability for justice-involved people in the aftermath of incarceration compared to their pre-incarceration status [28].

Despite the existence of re-entry assistance programs, housing is largely seen as being outside the purview of the justice system and there are no centralized agencies responsible for housing assistance [29,30]. Most formerly incarcerated people double up with relatives or friends until they are able to secure permanent housing [17,31–33]. This arrangement may be beneficial to some, as living arrangements that are in line with conventional social norms are more likely to motivate individuals to engage in responsible behavior and avoid deviant behavior [34]. For example, cohabitating with relatives or a spouse, but not a girlfriend, has been shown to be associated with less criminal activity [7,35,36].

However, the protective effect of living with relatives may be offset if it necessitates returning to a chaotic environment, a high-crime neighbourhood, or living within a social network that condones substance abuse or crime [37–41]. Returning to the neighbourhoods where drugs were obtained or taken also places formerly incarcerated individuals in an environment rich with drug cues that can trigger drug cravings [12,42,43]. For many, doubling up is not an option due to interpersonal conflict, lack of social support, or legal restrictions prohibiting formerly incarcerated people from residing with others who are in public housing, which leaves many formerly incarcerated people on the streets [44–46].

The impact of housing on formerly incarcerated individuals is pervasive and particularly salient for those in recovery. A study examining differential patterns of homelessness found that those with a specific constellation of risk factors including high substance use and arrest history were more likely to experience recurrent homelessness [47]. Current substance use has also been shown to predict future housing patterns. The results of a study of 400 homeless people in St. Louis, Missouri found only 18% of cocaine users were able to attain and retain stable housing in the following two years [48]. Research in the HIV risk and college drinking literature has also demonstrated strong evidence linking living arrangements, unstable housing, and substance use [49,50].

The research examining substance use across settings within the formerly incarcerated population is limited but essential to improving post-incarceration outcomes. The current study aims to address this gap in the literature by examining six months of retrospective housing and substance use data collected from a sample of formerly incarcerated individuals in recovery to answer the following research question: Which settings are associated with the most substance use? We predicted that settings with less oversight and financial obligation would have the highest proportion of participants exhibiting substance use and the highest proportion of time spent using substances while in that setting. As such, we expected homeless and precarious settings to have the highest substance use. The results of this study were expected to illustrate the importance of setting characteristics in substance use following incarceration and demonstrate the need for more comprehensive post-incarceration housing support to reduce the likelihood of relapse and recidivism outcomes.

## Methods

### Participants

A total of 270 adults (224 men and 46 women) were recruited from a large, Midwestern city for participation in a longitudinal, randomized study examining the impact of self-run recovery homes (i.e., Oxford House) on several indicators of adjustment, wellbeing, and recovery [51,52]. Most participants (n=251) were recruited from inpatient substance use treatment facilities while receiving treatment. The remaining participants were referred from case management/re-entry services (n=6) or inpatient substance use treatment facilities (n=13) but were not receiving services at the time of recruitment.

Inclusion criteria included being 18 years of age or older, in recovery from alcohol or drug dependence, and having been released from a correctional facility within the past 24 months. Participants who refused to participate in randomization or had violent crime or sex offense convictions (due to restrictions from one of the TCs) were excluded from the study. For the current study, participants who were missing housing or substance use data were excluded from the study, which yielded a sample size of 207.

### Procedure

Recruitment spanned from March 2008 to May 2011. Participants were randomly assigned into one of three treatment conditions (Oxford House, therapeutic community, usual aftercare) following informed consent. Baseline interviews were conducted at the recruitment sites, and the follow-up interviews were conducted on-site whenever possible. When on-site interviews were not possible, interviews were conducted over the telephone or in private locations. Occasionally it was necessary to conduct interviews in public locations (e.g., restaurants, libraries). Four follow-up interviews were conducted in six month intervals over a two-year period. The current study used data collected during the baseline assessment.

### Measures

#### Demographic Survey

A questionnaire generated by the researcher’s elicited information regarding race/ethnicity, gender, and age.

#### Timeline Follow-back

Alcohol and drug usage for the past 180 days was assessed using an adapted version of Miller and Del Boca’s (1994) Form 90 Timeline Follow-back. Participants were asked to mark important days and events on a 180-day calendar to facilitate recall of drug (yes/no) and alcohol (number of drinks) usage. Psychometric properties are favourable and have been validated with adult drug-abusing patients [53,54].

Similar to substance use assessment, living arrangements and housing stability for the previous six months were retroactively captured through a calendar adapted from the Residential Timeline Follow-Back Inventory [55]. Participants reported on the type of setting in which they lived, with whom they lived, whether they financially contributed toward their housing, and their reason for departure. Due to low response rate, items assessing living companions and reason for departure were omitted from analyses study. Residential mobility was determined by calculating the total number of moves within the six month period.

The eleven setting types were collapsed into four categories based on conceptual similarity (Table 1) to increase power and facilitate data analyses. The Regulated category included institutional settings with professional staff where substance use is prohibited or otherwise restricted. The Independent category included settings which likely offered more stability due to the participant’s financial contribution to the household.

The Precarious category included settings which likely offered less stability due to the lack of financial contribution to the household. The Homeless category was not composed of other condensed settings and was endorsed when participants were living in conditions that were not intended for housing. Due to the inability to determine the characteristics of the other setting, it was excluded from the four collapsed categories and coded as missing.

The psychometric properties for residential timelines and the aggregate categories have been established among homeless, substance using, and psychiatric populations [56,57].

#### Criminal history

Lifetime months of incarceration and history of criminal charges were assessed with the Addiction Severity Index Lite-CF (ASI-lite). Adapted from the Addiction Severity Index 5th Edition [58], the ASI-lite assesses seven potential problem domains in addition to demographic information. The following areas are evaluated: alcohol use, drug use, medical status, employment, legal, family and social relations, and psychiatric conditions. Questions assess lifetime and current (e.g., past 30 days) functioning. Test-retest reliability is excellent composite scores (≥ 83) [58].

### Analytic Plan

To examine the association between housing and substance use over time, the 180 data points for the three timelines (alcohol use, drug use, housing setting) were matched for each participant and entered into an Excel 2010 spread-sheet. A series of formulas were used to calculate the number of day’s alcohol and drugs were used within each of the eleven settings for each participant. The data were then imported into SPSS v21 for statistical analyses.

New variables were created that collapsed the eleven housing settings into four, conceptually distinct categories. To account for different lengths of time spent in each setting, variables were created to capture the proportion of time alcohol and drugs were used in each category. Means of proportion of use were also calculated across categories.

## Results

### Sample characteristics

Most of the participants were male (83.1%) and never married (77.1), with a mean age of 40.31 (SD=9.75) years and 10.83 (SD=1.99) years of education. The ethnic distribution of the sample was 71.0% African American, 23.2% White, and 4.3% Latino, and <2% Native American or multi-racial. Regarding legal involvement, most participants reported a history of non-violent criminal charges, including public order (83.6%), drug (73.9%), and property (69.1%) crimes, with only a third (36.7%) reporting violent criminal charges. They had been incarcerated an average of 9.36 (SD=18.45) times with the most recent incarceration lasting an average of 14.27 (SD=16.19) months and time since most recent incarceration release 144 (SD=122.20) days. Heroin/ opiates were the most endorsed substance of choice (44.4%), followed by crack/cocaine (24.2%), alcohol (16.4%), marijuana (7.2%), polysubstance use (6.3%), and amphetamine/crystal methamphetamine (0.5%). Participants had been treated on average 0.49 (SD=1.28) times for alcohol use and 2.62 (SD=2.93) times for illicit substance use problems.

### Housing and substance use

Table 2 presents data on the housing and substance use patterns of the sample in the 180 days prior to the baseline assessment of the longitudinal study (Jason et al.).

#### Housing

On average, participants moved 1.72 (SD=1.10) times in the previous six months, with nearly the entire sample (98%) having spent time in Regulated settings; more than half of the sample (66%) was released from a correctional facility after spending an average of 95 days incarcerated. Three quarters (76%) received inpatient residential substance use treatment and 18% went through detox in a medical facility. Nearly half (45.4%) of the sample lived in Precarious settings, with only a small proportion (10.2%) having lived in independent settings where they financially contributed to the household. Literal homelessness was experienced by 16.4% of the sample for an average of 55 days.

#### Substance use in settings

**Figure 1:**
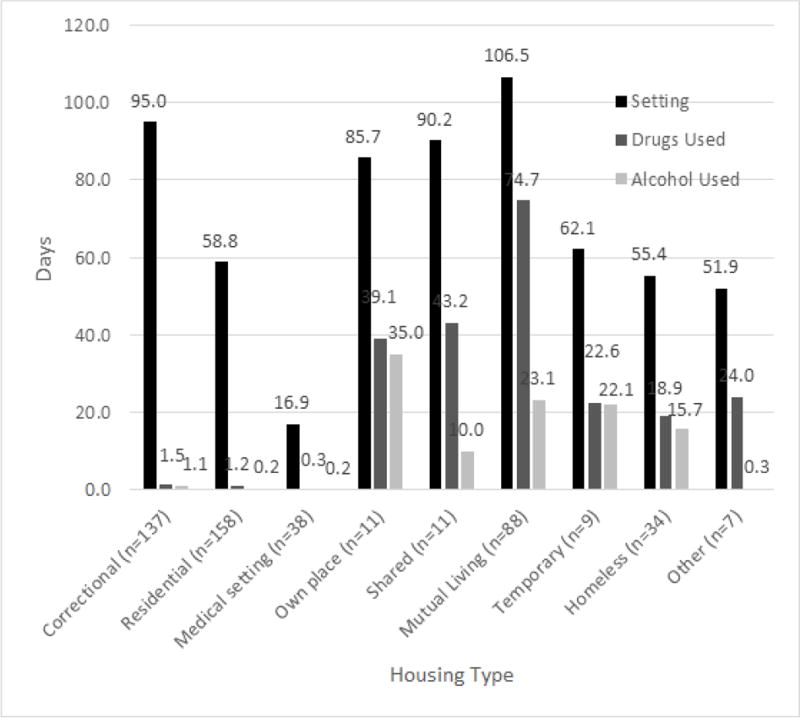
Average days spent in setting compared to average day’s drug and alcohol use in setting. The precarious settings (mutual living, temporary) are associated with the highest proportion of time spent using substances while in the setting.

Table 2 provides a detailed overview of the substance use within each of the settings. More than half of the sample (65.7%) reported using either drugs or alcohol during the previous six months, with drugs being used more often than alcohol across settings (59.9% versus 30.0%). Substance use was reported across all settings; a test of proportions found a significantly lower proportion of participants in Regulated settings (28.6%) used any substance compared to the remaining superordinate categories (Independent Z=−6.08, p<0.001; Precarious Z=−9.89, p<0.001; Homeless Z=−5.38, p<0). There was also a significantly higher proportion of participants engaged in substance use when living in Precarious settings compared to the Homeless category (90.4% *vs*. 76.5%; Z=2.05, p=0.040), which was driven by the higher proportion of participants who reported using drugs in the Precarious category (83.0% *vs*. 64.7%; Z=2.21, p=0.027). Figure 1 illustrates the magnitude of alcohol and drug use as measured by the number of days substances were consumed relative to the time spent in the setting. Participants spent significantly less time using alcohol and drugs in the Regulated setting compared to the other settings (average proportion of alcohol use: Independent Z=−5.73, p<0.001; Precarious Z=−5.95, p<0.001; Homeless Z=−6.11, p<0.001; average proportion of drug use: Independent Z=−7.99, p<0.001; Precarious Z=−11.66, p<0.001; Homeless Z=−6.65, p<0.001). Bivariate comparisons examining the average proportion of time spent using alcohol and drugs among the remaining three settings revealed one significant difference in drug use between the Precarious and Homelessness categories (Z=2.90, p=0.004), whereby those in the Precarious setting spent twice as much time using drugs compared to those in the Homeless setting.

## Discussion

The findings of our exploratory study were consistent with previous research that has shown the majority of formerly incarcerated individuals have unstable post-incarceration housing outcomes. Furthermore, the current study revealed a strong association between housing and substance use whereby settings with less oversight and financial obligation (e.g., couch-surfing, homelessness) were associated with the most substance use. Of note, substance use was reported across all settings, including correctional facilities. Although Regulated settings had a significantly lower proportion of participants using and a lower frequency of usage compared to other settings, the use was still markedly higher than would be expected given the security of these places; over a quarter of participants who were in a correctional facility reported substance use. Thus the temptation of substances is present in even highly regulated settings.

The other notable finding was regarding the substance use in Pre-carious settings. Given the high association between substance abuse and homelessness [47,48], it is surprising that drug use was significantly higher in the Precarious settings (most notably Mutual Living). This discrepancy may be due to increased availability of substances from others in the household and more disposable income for substances given the lack of financial contribution to the household. The high substance use within Precarious settings is perhaps the most alarming finding of the study, as nearly half the sample were living in these arrangements at some point in the previous six months. Of note, Independent settings also appeared to exhibited high substance use risk; however, effects were not found due to low statistical power.

The current study entails the secondary analysis of existing data; thus, several limitations should be considered when interpreting the study findings. Although the study includes several data points across a six-month period, it was collected retroactively at one time and data were analyzed in aggregate. As a result, causality, individual change, and timing effects were not examined. Furthermore, the associated substance use within each of the settings is not independent from the other settings, as most participants lived in multiple settings (and conversely, substance use data were not available across all settings for every participant). Data regarding setting characteristics that may have influenced housing and substance use, such as information regarding other people in the setting, were incomplete or unavailable and therefore not analyzed in this study. Additionally, bivariate associations examining substance use and setting did not include confounding factors that may have contributed to the observed effects. Finally, the power to detect true effects in certain subgroups (Independent, Homeless) was low due to a small sample size.

The findings and limitations in this exploratory study suggest several avenues for future research. Longitudinal studies that analyze within and between group differences designed with the primary purpose of examining housing and associated substance use upon institutional release may help elucidate this complex relationship. For example, the original study from which the data for the current study were derived [51,52] randomly assigned formerly incarcerated individuals in recovery to three conditions following residential substance use treatment: usual care (where they would naturally stay after completing treatment including staying with friends or family, their own place, homeless shelters, etc.), therapeutic communities, and Oxford Houses (self-run, abstinent recovery homes). The results indicated that longer lengths of stay in the therapeutic communities and Oxford Houses were associated with decreased substance use; however, this study did not distinguish substance use among the typical, non-recovery settings in which most formerly incarcerated people find themselves. Although randomization of multiple settings may not be feasible, a longitudinal observational study would allow researchers to control confounding variables, map risk trajectories, and identify the characteristics of individuals at greatest risk.

The study findings call attention to the limited housing options formerly incarcerated individuals encounter in the aftermath of institutional release and the impact housing may have on recovery. In addition to the difficulty of establishing resources necessary to function independently while living in unstable housing (e.g., employment, transportation, legal obligations), formerly incarcerated individuals with substance use disorders also have the additional challenge of maintaining sobriety. To prevent relapse and recidivism, more resources should be allocated to help transitioning individuals establish long-lasting stability in the community.

## Figures and Tables

**Table 1:** Housing settings and aggregate categories.

Assigned Category	Residential Living Settings
Regulated	Correctional facility (prison, jail)
	Residential program with staff
	Medical setting (e.g., detox, medical hospital)
Independent	House/apartment (living in own place)
	Shared housing (financially contributing)
Precarious	Mutual living (living in someone else’s home but providing little or no set financial contribution)
	Temporary (e.g., couch surfing, hotel room)
Homeless	Literal homelessness (e.g., car, bus station, park, shelter, etc.)
Missing	Other

**Table 2:** Summary of living arrangement characteristics and substance use by setting for 180 day period prior to baseline.

Setting	Lived in Setting^1^	Avg Days in Setting^2^	Used Any Substance in Setting^2^	Used Drugs in Setting^2^	Used Alcohol in Setting^2^	Avg Proportion of Drug Use in Setting	Avg Proportion of Alc Use in Setting
	n (%)	M (SD)	n (%)	n (%)	n (%)	M (SD)	M (SD)
Regulated	202 (97.6)	37.37 (22.40)	58 (28.6)	52 (25.6)	23 (11.3)	.02 (.08)	.01 (.05)
Correctional	137 (66.2)	94.98 (51.08)	36 (26.3)	31 (22.6)	13 (9.5)	.04 (.14)	.02 (.12)
Residential	158 (76.3)	58.78 (49.71)	22 (13.9)	21 (13.3)	11 (8.0)	.02 (.06)	.01 (.02)
Medical	38 (18.4)	13.74 (29.67)	5 (13.2)	5 (13.2)	2 (1.5)	.06 (.21)	.02 (.12)
Independent	21 (10.15)	46.07 (22.47)	20 (95.2)	17 (81.0)	9 (42.9)	.46 (.37)	.24 (.34)
Shared Housing	11 (5.3)	90.18 (46.36)	11 (100)	10 (90.9)	5 (45.5)	.47 (.34)	.17 (.22)
Own house/Apt	11 (5.3)	85.72 (40.98)	10 (90.9)	8 (72.7)	5 (45.5)	.49 (.43)	.38 (.45)
Precarious	94 (45.4)	52.83 (26.85)	85 (90.4)	78 (83.0)	36 (38.3)	.61 (.38)	.20 (.33)
Mutual Living	88 (42.5)	106.51(51.74)	81 (92.0)	76 (86.4)	32 (36.4)	.65 (.36)	.20 (.34)
Temporary	9 (4.3)	62.11 (72.76)	6 (66.7)	4 (44.4)	6 (66.7)	.22 (.40)	.24 (.29)
Homeless	34 (16.4)	55.41 (56.25)	26 (76.5)	22 (64.7)	16 (47.1)	.32 (.36)	.24 (.34)
Any setting			136 (65.7)^1^	124 (59.9)^1^	62 (30.0)^1^	.24 (.31)	.09 (.21)

**Note:**

1 Percentage of sample size n=207;

2 Percentage of participants having lived in that setting.
